# P39 Primary antiphospholipid syndrome before development of severe systemic lupus erythematosus – a case report

**DOI:** 10.1093/rap/rkae117.070

**Published:** 2024-11-05

**Authors:** Asma Ibrahim, Gouri Koduri

**Affiliations:** Mid and South Essex NHS Foundation Trust, Southend, United Kingdom; Mid and South Essex NHS Foundation Trust, Southend, United Kingdom

## Abstract

**Introduction:**

Antiphospholipid syndrome (APS) has long been associated with systemic lupus erythematosus (SLE). Graham Hughes first reported APS in 1983 in cohorts of SLE patients with recurrent thrombosis. We present a case of a young man who developed severe manifestations of SLE 7 years after the diagnosis of primary APS and we look at the literature for predictors in primary APS to develop into SLE.

**Case description:**

A 28 year old Caucasian male with Turkish ancestry was diagnosed with triple positive primary APS at the age of 21 when he presented with bilateral pulmonary embolisms. He was stable on warfarin with target INR between 3-4. He had been having occasional mouth ulcers, lethargy, mild paraesthesia of his feet and headaches for 2 months. He then presented in February 2024 with short history of pleuritic chest pain and marked breathlessness. Initial cross sectioning imaging did not reveal pulmonary embolism. A week later, echocardiogram demonstrated large global pericardial effusion and small pleural effusions. Ejection fraction was preserved. Interval MRI scan showed pericarditis without evidence of myocarditis. Almost 600 mls of heavily blood stained pericardial fluid was drained. Analysis of the pericardial fluid showed inflammatory processes with macrophages, neutrophil and lymphocytes. Culture was negative including for tuberculosis and cytology negative for malignancy. Immunology revealed ANA 1:160, ENA normal, dsDNA was initially negative but upon interval testing was high at 27.5 IU/mL, IgG 13.1 g/L, C3 1.2 g/L, C4 <0.1 g/L, CRP 143, ESR 30. He had anaemia, lymphopenia and positive direct antiglobulin test. ANCA and rheumatoid factor were unremarkable. Renal and joints were unaffected. Due to his Turkish ancestry, he was extensively investigated for the possibility of Behcet’s with normal femoral vein ultrasound and negative HLA-B51 genotype. His manifestations fulfilled the SLICC criteria to be diagnosed with SLE. His response to intravenous methylprednisolone infusions and subsequent oral steroid was marked. He was initiated on dual treatment of hydroxychloroquine and mycophenolate mofetil and is currently recovering.

**Discussion:**

We relooked at this immunology when he was first diagnosed with primary APS in 2017. He was strongly positive for ANA at titre of 1:640 but did not have manifestations of SLE then. We researched literature on the predictors for primary APS to develop into SLE. Both disease spectrums have several overlapping manifestations including haemolytic anaemia, thrombocytopenia, lymphopenia, neurological symptoms, renal manifestations and livedo reticularis.

Freire *et al* (1) retrospectively analysed 80 patients with primary APS and found 14 (17.5%) progressed to develop APS-SLE in 5.2 +/- 4 years. ANA positivity was found in 100% those who eventually developed APS-SLE. Other specific autoantibodies including anti-dsDNA, anti-ribosomal P, anti-Ro, anti-La and anti-U1RNP are found more commonly in the cohort too. In their APS-SLE cohort, they also found a correlation with younger age at the time of diagnosis of APS and the duration of the disease.

Andreoli L *et al* (2) demonstrated anti-nucleosome antibody (anti-NCS) was frequently found in their cohort of APS-SLE patients but no relationship between clinical-serological was found, except for a weak correlation between anti-dsDNA antibodies. 2 of their primary APS who developed severe SLE had displayed high titres of anti-NCS years before. Tarr T *et al* (3) found 4 of their primary APS who developed SLE all presented with cerebrovascular events as the thrombotic manifestation.

**Key learning points:**

• Primary APS has been associated with the development of SLE

• Certain antibody profile such as ANA, anti-nucleosome antibody may predict the development of SLE in primary APS patients

• Younger age, cerebrovascular events as thrombotic manifestation at diagnosis of primary APS and the length of duration of primary APS may be associated with the development of SLE at a later stage

